# The Nordic Nutrition Recommendations 2022 – handbook for qualified systematic reviews

**DOI:** 10.29219/fnr.v64.4404

**Published:** 2020-06-18

**Authors:** Erik Kristoffer Arnesen, Jacob Juel Christensen, Rikke Andersen, Hanna Eneroth, Maijaliisa Erkkola, Anne Høyer, Eva Warensjö Lemming, Helle Margrete Meltzer, Þórhallur Ingi Halldórsson, Inga Þórsdóttir, Ursula Schwab, Ellen Trolle, Rune Blomhoff

**Affiliations:** 1 Department of Nutrition, University of Oslo, Oslo, Norway; 2 Norwegian National Advisory Unit on Familial Hypercholesterolemia, Oslo University Hospital, Oslo, Norway; 3 National Food Institute, Technical University of Denmark (DTU), Kgs. Lyngby, Denmark; 4 The Swedish Food Agency, Uppsala, Sweden; 5 Department of Food and Nutrition, University of Helsinki, Helsinki, Finland; 6 The Norwegian Directorate of Health, Oslo, Norway; 7 Department of Environmental Health, Norwegian Institute of Public Health, Oslo, Norway; 8 School of Health Sciences, University of Iceland, Reykjavík, Iceland; 9 Department of Medicine, Endocrinology and Clinical Nutrition, Kuopio University Hospital, Kuopio, Finland, and Institute of Public Health and Clinical Nutrition, University of Eastern Finland, Kuopio Campus, Kuopio, Finland; 10 Division of Cancer Medicine, Oslo University Hospital, Oslo, Norway

**Keywords:** dietary reference values, food-based dietary guidelines, systematic reviews, national food and health authorities, evidence-based nutrition, nutrient recommendations, Nordic and Baltic countries

## Abstract

**Background:**

Systematic reviews (SRs) constitute a major part of the Nordic Nutrition Recommendations (NNRs). The step-by-step procedure used to develop SRs has evolved considerably over time and is often tailored to fit the exposure and outcomes in focus.

**Objective:**

To describe a detailed procedure for developing qualified SRs commissioned by the NNR2022 project.

**Design:**

Scrutinizing procedures of recent SRs commissioned by leading national food and health authorities or international food and health organizations.

**Results:**

The following eight steps must be included when developing qualified SRs for the NNR2022 project: 1) define research question, 2) protocol development, 3) literature search, 4) screening and selection of studies, 5) data extraction, 6) assessing risk of bias, 7) synthesis and grading of total strength of evidence, and 8) reporting according to certain standards.

**Discussion:**

This guide is based on the guidelines developed for the fifth edition of NNR but includes some important new domains in order to adhere to more recent, authoritative standards.

**Conclusion:**

All qualified SRs in the NNR2022 project will follow the protocol described here.

## Popular scientific summary

- The process to develop systematic reviews (SRs) requires a step-by-step predefined approach.
- This article describes a detailed procedure for developing qualified SRs in the Nordic Nutrition Recommendations 2022 (NNR2022) project.
- The new protocol represents an important update compared to corresponding guide developed for the fifth edition of the NNR.

This article is the third of a three-part series for the Nordic Nutrition Recommendations 2022 (NNR2022):

1. *Principles and methodologies (1)*,
2. Structure and rationale of qualified systematic reviews (2), and
3. *Handbook for qualified systematic reviews* (this article).

Together, these documents constitute a comprehensive and precise framework for how NNR will be updated. This article is a guide on how to conduct systematic reviews (SRs). The first paper describes the principles and methods used for developing the sixth edition of the NNR (NNR2022). The second paper, *a priori,* discusses and describes the structure and rationale for the SR methodology used in the NNR2022 project.

**Fig. 1:**
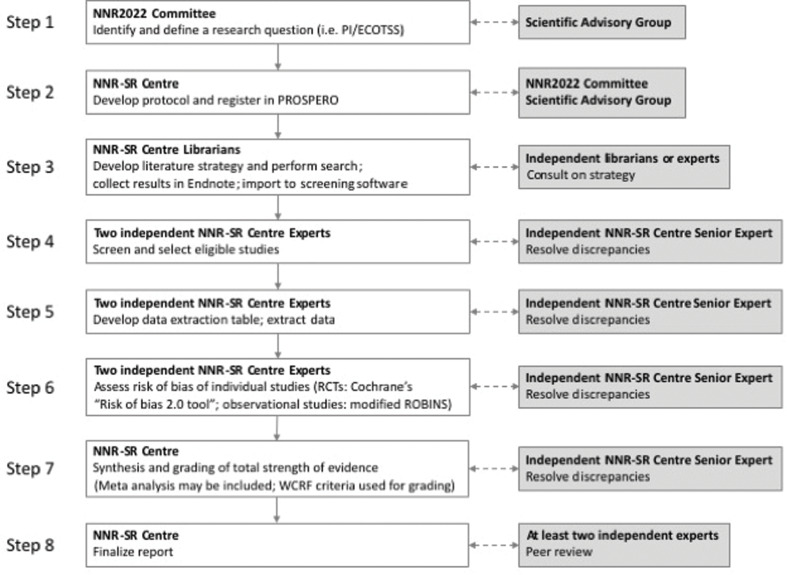
The individual steps involved when conducting SRs in NNR2022 project.

The NNR-SR Centre will follow established, state-of-the-art criteria for evaluating the methodological quality of the included studies and the overall strength of the scientific evidence (Fig. 1). Transparency wil l be ensured through detailed documentation of the decision-making process. All SRs will be evaluated by external reviewers. Also, a scientific reference group will be assigned to the project and will be consulted for general overview and on specific matters.

This handbook is based on a corresponding guide developed for NNR2012 (3) but includes some important new domains in order to adhere to more recent, authoritative standards, as detailed in the companion paper on SRs (2).

## Step 1: Research question

### Identifying and defining the research question(s)

It is important to identify the most important research questions, to pose questions that are possible to answer, and to define criteria for inclusion and exclusion of studies. To enable a focused literature search, the research questions should be formulated so that they can be answered with data from observational or experimental human studies. In some cases, animal studies and cellular and molecular studies may also be relevant. Here, it is useful to develop an analytical framework, for example, visual maps outlining relationships within the target population with respect to exposures, modifying factors, biological effects of a nutrient/food component, and outcomes of interest. The framework will facilitate the identification and definition of appropriate research questions (4).

The Population, Intervention (or exposure), Comparator, outcome(s) (PICO) approach is commonly used to formulate research questions related to the causal effect of an exposure on a health outcome (Box 1). The following components should be defined for all the research questions: population/participants, intervention/exposure, control, outcome, time frame, study design, and settings ( PI/ECOTSS).

Box 1 Components of the research question. Example from an intervention study

**Table:** 

Population/participants	Intervention/exposure	Control/comparison	Outcome	Time frame	Setting	Study design
Description of the study population, e.g. Age Sex Diagnosis Life style factors Socioeconomic characteristics Other risk factors Other diseases	Definition and description of the methods Intervention procedure Dietary modification (e.g. use of ‘normal foods’) Minimum duration Compliance Relevance to Nordic population	Definition and description of the method for controls Placebo Other treatment No treatment	Outcome measures related to the individual, such as survival, quality of life, illness or change in symptoms, well-established risk factors and markers. Also, complications or side-effects of the intervention should be considered.	Duration of intervention and/or follow-up	Background context, co-interventions, healthcare etc.	Randomized controlled trial
Example for an interventional study						
Healthy adults	Altered dietary macronutrient composition	Usual dietary macronutrient composition	Body weight Body fat Waist circumference	≥1 year	Free-living	Randomized controlled trials

The example PI/ECOTTS shown in Box 1 could be ‘translated’ into a research question (adapted from Ref (5): ‘what is the effect of different dietary macronutrient composition on long-term (≥1 year) change in weight/body fat in a healthy, adult population?’

Dietary modifications may involve one or several components of the diet. However, it should be clearly defined which components are being studied, and how (methodology).

## Step 2: Develop a protocol

Before conducting the SR, a protocol must be developed. All protocols for the NNR SRs will be registered in advance in the PROSPERO database (http://www.crd.york.ac.uk/prospero). The protocol should specify the following (6–10):

1. The problem definition/research question(s), rationale, and objectives. Refer to PI/ECOTSS, with descriptions of study population, outcomes, intervention or exposure, and type of study designs.
2. The planned search strategy:
  - Time frame to be considered
  - Databases to be searched, and other sources if relevant (e.g. reference lists of articles and citation searches)
  - Any efforts to obtain unpublished data (e.g. contact with study authors)
3. Eligibility criteria
  - Again, refer to PI/ECOTSS, time frame, and publication status. Reasons for including one or several types of study designs should be explained. Restrictions of specific publication types should also be justified.
  - Describe clearly the nature of the interventions/exposures and outcomes of interest. Definitions and assessments of exposure and endpoints should be specified.
4. The selection process, including who will screen titles/abstracts and full-text papers, and how disagreements will be resolved.
5. Methods for managing and extracting data from studies.
  - List all variables for which data will be extracted.
  - Include data extraction form(s).
6. A plan for assessing the risk of bias (RoB) in individual studies, including which tools to use (2), how they will be implemented, and criteria for scoring low or high RoB. Describe how results of the risk-of-bias assessment will contribute to the synthesis and overall findings of the review.
7. A plan for synthesizing the results; whether a quantitative synthesis (meta-analysis) is planned, and criteria for when to conduct meta-analyses, methodology for meta-analyses (e.g. statistical models and choice of effect measure), and how heterogeneity will be assessed. Indicate how narrative syntheses of the evidence will be presented (e.g. what will be included in tables or text only), and in what order (e.g. by research question and outcome).
8. Any planned subgroup analyses and potential effect modifiers to be assessed (if the necessary data should be available).
9. Intended methods to evaluate the quality/strength of evidence and to summarize the findings from the review.

## Step 3: Literature search

Planning the search strategy (Box 2) and performing the search will require the involvement of the research librarian and/or other information specialists with experience in SRs. It is also advisable that the search strategy is evaluated by an independent expert/research librarian before the final search is performed.

Box 2 Key standards for literature search

A comprehensive, systematic, and sensitive literature search is a fundamental and critical domain of systematic reviews. Search strategies for each research question will be planned and included in the protocol (see above), including:

1. The time period to be considered
2. Which databases to be searched, and other sources, if relevant (e.g. reference lists of articles), citation searches
3. Use of any search filters, indexing terms, and free text terms
4. Efforts to obtain unpublished data (e.g. contact with study authors), if needed

In general, terms related to study design should not limit the search, as they (especially non-randomized study designs) may not be reliably or consistently indexed in the databases (13, 14). However, if limitations on study design are necessary and justified, the highly sensitive, validated search filter by Cochrane is recommended for identifying randomized trials, which in PubMed consists of the following:

#1 randomized controlled trial [pt]
#2 controlled clinical trial [pt]
#3 randomized [tiab]
#4 placebo [tiab]
#5 drug therapy [sh]
#6 randomly [tiab]
#7 trial [tiab]
#8 groups [tiab]
#9 #1 OR #2 OR #3 OR #4 OR #5 OR #6 OR #7 OR #8
#10 animals [mh] NOT humans [mh]
#11 #9 NOT #10

Validated search filters are also available from SIGN (15), which may be considered (if used, these must be reported), for example, for observational studies in MEDLINE:

#1 Epidemiologic studies/
#2 Exp case control studies/
#3 Exp cohort studies
#4 Case control.tw.
#5 (cohort adj [study or studies]).tw.
#6 Cohort analy$.tw.
#7 (Follow up adj [study or studies]).tw.
#8 (observational adj [study or studies]).tw.
#9 Longitudinal.tw.
#10 Retrospective.tw.
#11 Cross sectional.tw.
#12 Cross-sectional studies/#12 Or/1-12

Articles indexed as ‘letters’, ‘letter to the editor’, or ‘research letters’ should also be included since these may contain relevant data or reports from individual studies. Any other prespecified restrictions regarding publication type must be justified.

### Search strategy

The purpose of the search strategy is to find the best and most relevant evidence available. Finding all evidence is impossible, but the searches should be highly sensitive, which implies that precision will be reduced. The search syntax should include both indexing terms (‘subject headings’, e.g., MeSH terms in MEDLINE) and free text terms. All synonyms and spelling variants should be included, and appropriate Boolean operators and truncations are to be used. It is important to have a list of known key publications that should be identified in the search, to check whether the search strategy is sensitive enough (11).

To construct an effective set of search terms, the research question should be divided into components according to the PI/ECOTSS approach (population/participants, intervention/exposure, control, outcome, time frame, setting, and study design). However, all of the PI/ECOTSS elements are not necessary to include in the search, as including all these will reduce sensitivity, and they may not be adequately indexed in the databases. The search should not restrict outcomes and should not include any language restrictions. Only studies in English, Nordic or Baltic languages will, however, be considered.

### Selection of databases

MEDLINE (PubMed), EMBASE, and the Cochrane Library are the main information sources in the field of nutrition and medicine. Other databases and online hosts, such as Web of Science, or subject-specific databases, may be considered. At least two databases must be searched, and these should always include MEDLINE and Cochrane’s CENTRAL database.

Another source that should be searched is the reference lists of the already retrieved articles. A forward citation search (or *citation tracking*) from included studies, in citation indexes such as Web of Science, Scopus, or Google Scholar, should also be performed. This will identify potentially highly relevant new papers citing the references retrieved from the initial search and included in the review (12).

Searching for ‘grey literature’ (e.g. non-peer-reviewed scientific papers outside of the mentioned databases such as conference abstracts, theses, and clinical trial registries) is not mandatory. These sources may identify publication or selective reporting bias, but the methodological quality may be uncertain (13–15). Study authors or study sponsors may be contacted to clarify uncertainties about eligibility, outcomes, or other unpublished data; these should be referred to in the report.

### Study design

Study types that may be included in the systematic literature reviews are randomized and non-randomized controlled intervention studies and prospective and retrospective cohort studies. This includes studies assessing disease outcomes, net losses of nutrients, nutrient balances, factorial calculations, etc. Multifactorial intervention studies including diet and physical activity will also be considered. Retrospective case-control studies, where the measure of exposure occurred after or concurrent with the outcome, will only be used when results from other study types are not available. Cross-sectional studies will be used for describing prevalence, and animal studies will only be used to describe mechanisms.

### Managing search results

The search results will be imported to a reference managing program (e.g. EndNote), where duplicate records are removed, before importing the list of studies into software for screening, such as *Rayyan* (see below), for the screening of titles and abstracts.

The search strategy used, and bibliographic databases searched, will be clearly documented. This makes it possible for others to reproduce the SRs, allows comparison across reviewers, and serves as a foundation for an efficient updating of the systematic review as new findings emerge. The full search strategies for each database will need to be included in the review. The search strategies must be copied and pasted, not re-typed, exactly as run and reported in full, together with the search set numbers and the number of records retrieved. The number of unique records retrieved will also be reported in the results section of the review under the heading ‘Results of the search’ in a flow diagram.

The final search should be performed no later than 12 months before the review is published. The exact date for this should be reported in the SR report’s methods chapter.

## Step 4: Screening and selection of studies

Articles retrieved from the search, after duplicates are removed, will be evaluated for relevance and suitability for inclusion in the review by assessing the study characteristics in relation to the research question(s). The selection of papers has two phases: first, a screening of records’ titles and abstracts to exclude obviously irrelevant papers, and second and subsequently, a full text reading of the potentially relevant papers (Box 3).

Box 3 Key standards for screening and selection of studies

- Screening and selection of studies for inclusion/exclusion is performed by at least two members of the team, working independently.
- Initially, titles and abstracts of all studies will be screened, and then full texts of potentially eligible articles.

The inclusion or exclusion of studies should not be affected by the reviewers’ knowledge of the study results, should be reproducible, and should be independently undertaken by pairs of experts. Studies will therefore be included or excluded according to the prespecified (in the protocol), unambiguous *eligibility criteria* outlined by the NNR2022 Committee in collaboration with the NNR-SR Center.

### Eligibility criteria

The PI/ECOTSS components define much of the eligibility criteria for selecting the studies. Additional criteria may include dose levels (plausibility at dietary level), minimum number of subjects per study arm, background diets, baseline nutritional status, minimum study period, and statistical analysis (Box 4).

Box 4 Examples of aspects that may be considered when defining eligibility criteria

Examples of aspects that may be considered when defining eligibility criteria (must be justified and predefined in the protocol):

- Diseased or healthy subjects only
- Intervention/exposure and comparator of interest
- Outcome measure
- Type of study
- Number of participants – study size
- Dietary assessment methods
- Publication type (original articles, conference abstracts, etc.)
- Time period for publication
- Length of follow-up
- Length of intervention period
- Other simultaneous interventions
- Comorbidity
- Previous interventions

It is important that the eligibility criteria do not introduce bias. They should be guided by the analytic framework, taking potential effect modification into account. If studies are excluded based on lack of outcome data, it should be ensured that the outcome was not measured in the study, or simply not reported. Any changes of the eligibility criteria post hoc must be reported in the review.

The primary target population for NNR2022 is defined as the general healthy population. This means that studies focusing only on treatment of patients with overt diseases should generally be excluded. Studies involving subjects with increased metabolic risks or pre-disease states, for example, with established risk factors, will be considered. Studies including both healthy populations and people with elevated risk of chronic disease or established chronic disease, including obesity, hypercholesterolemia, and hypertension, may also be included if deemed appropriate.

The minimum acceptable duration/follow-up time of the studies should be specified in the protocol. As a rule of thumb, intervention studies should be at least 4 weeks, but specific criteria should be defined depending on the type of outcome risk factor versus disease endpoint. Observational (prospective) studies should be at least for 6 months; longer periods may be required depending on dietary exposure and outcome. For all the study types, the intake ranges for nutrients and dietary sources should be relevant to the Nordic setting.

### Selection process

The screening of titles and abstracts may be guided by a checklist based on the PI/ECOTSS and the predefined eligibility criteria. The eligibility criteria should be listed according to priority, and the eligibility assessments follow this order, for increased efficiency. For instance, when the most important exclusion criterion is identified first, the other criteria do not need to be assessed. In this phase, the screeners should be ‘overinclusive’. RoB is not an exclusion criterion as it will be assessed in a later step.

### Pilot testing of screening phase

The eligibility criteria should be pre-tested on the first 10% of titles/abstracts. Inter-rater agreement may be calculated; a kappa statistic (k) between 0.4 and 0.59 is considered fair, a value of between 0.6 and 0.74 is considered good, and a value of >0.75 is considered an excellent agreement (16) (see, e.g., Cochrane Handbook 7.2.6.1 at http://handbook-5-1.cochrane.org for calculations). Note that this should only be done in the pilot testing phase to identify problems or misunderstandings, and not be used as a standard, as the statistic does not say anything about the effect of disagreements on the results. Discrepancies, that is, k < 0.6, will be assessed and used to clarify or refine eligibility criteria and/or research question, if relevant, together with the NNR2022 Committee and the NNR-SR Center. In the formal screening process, discrepancies will be resolved by consensus. The screening should be reliable and comprehensive, but the aim is not perfect agreement.

Both selection phases are carried out by at least two independent members of the review team to minimize subjectivity and random errors. If at least one of the assessors votes for inclusion, the paper will pass to full-text screening. When both members vote for exclusion, it will be excluded without further discussion. The protocol should specify how discrepancies between reviewers in the full-text screening phase will be resolved; in general, it is done by a discussion with a third (senior) team member or the whole team until consensus is reached. If necessary, correspondence with the primary research authors should be attempted to clarify the study eligibility or lack of important data. The free of charge web service *Rayyan* is recommended for managing the screening of titles and abstracts (17).

The decisions must be documented and noted in a flow diagram, specifying the number of papers/studies assessed and remaining at each step in the process, and the number of excluded papers (see the Preferred Reporting Items for SRs and Meta-Analyses guidelines (PRISMA) and the PRISMA Flow Diagram Generator) (18, 19). Papers excluded after full-text reading will be listed in a separate table, including at least one reason for exclusion. If any full-text paper is impossible to obtain, this should also be noted as a reason for exclusion.

Individual studies are often reported in multiple publications, for example, with different endpoints or follow-up times. On the other hand, the unit of interest in the SR is the overall study, and not the specific paper. It is important that multiple publications from the same studies are linked together and not double-counted. Some papers may also report multiple studies, which should be counted accordingly. Identifying duplicate articles based on the same study is not always straightforward, but some helpful hints are (16):

- Study authors
- Place and setting
- Specific details about interventions (e.g. dose and frequency)
- Number of participants and baseline data
- Dates and duration

The search and selection processes must be documented as they proceed. Studies excluded after full-text reading must also be reported in separate tables, though not obviously ineligible studies excluded from the initial screening. The reasons for exclusion of each potentially relevant study must be stated explicitly.

## Step 5: Data extraction

The prespecified data will be collected into pre-made *extraction* forms that will be used later in the analyses (Box 5). The extraction forms should be pilot tested.

Box 5 Key standards for data extraction

- A standardized data extraction form is created for each systematic review (SR).
- The study is the unit of interest; duplicate papers from the same study are combined.
- Data from the included studies are extracted by at least two reviewers working independently.

The tables will give the reader the possibility to judge the reliability of the conclusions and should include information on the source reference, research question, methods, study design, implementation, results, and methodological quality. The exact forms will vary from review to review, but there will be many similarities. The PI/ECOTSS should be described in detail in ‘evidence tables’ and can easily be adapted for the specific review. An evidence table is a comprehensive compilation of *a priori* defined data elements extracted from the primary studies that are judged to be important in the interpretation of the evidence. Table 1 gives an example on evidence table headings providing some of the main relevant data elements.

**Table 1:** Example on evidence table headings (3)

Reference details First author Year Country	Study design (RCT, cohort, etc.)	Population, subject characteristics Inclusion/exclusion criteria Setting No. at baseline Male/Female Age Ethnicity of the subjects	Outcome measures Disease, biological measures	Intervention/exposure	Time between baseline exposure and outcome assessment	Dietary assessment method FFQ, food records Internal validation (y/n) (see separate table for more details)	No of subjects analyzed	Intervention Intervention (I) (dose interval, duration) Control (C) (active, placebo, usual care, etc.) Compliance, achieved dietary change, adherence to dietary targets, actual dietary change	Follow-up period, drop-out rate (from baseline to follow-up, or from end of intervention to follow-up) Drop out (%)	Results Results (I, C) (Absolute difference, RR, OR, *P*-value, confidence interval, sensitivity, specificity, observer reliability, etc.)	Confounders adjusted for	Study quality (risk of bias)	Comments

For later quantitative syntheses, the most detailed numerical data should be collected. In multi-component trials, only the groups fulfilling the eligibility criteria should be included in the data extraction tables. It is recommended to seek out non-reported data from the investigators.

The following study characteristics (Table 2) should also be considered, if relevant for the research question (adapted from the Cochrane Handbook (16)).

**Table 2:** Study characteristics

	Data to extract
Methods	Study design Total study duration Sequence generation Allocation sequence concealment Blinding Other concerns about bias
Participants	Total number Setting Diagnostic criteria Age Sex Country Comorbidity Socio-demographics Ethnicity Date of study
Interventions	Total number of intervention groups Specific intervention Intervention details Integrity of intervention
Outcomes	Outcomes and time points collected and reported Outcome definition (e.g. diagnostic criteria) Unit of measurement For scales: upper and lower limits, whether high or low is good
Results	Number of participants per intervention/exposure group Sample size for each outcome Missing participants for each outcome Summary data for each intervention group Estimate of effect, with confidence interval, *P*-value Subgroup analyses
Other	Funding source Key conclusions by study authors Miscellaneous comments from study authors References to other relevant studies Correspondence Miscellaneous comments from review authors

Outcome data should be extracted in the format as reported. Sample size should be collected for each end point data and for the overall study.

Food/nutrient/supplement intake information might include intake/dose, source of nutrient, mode of administration (supplement/foods), and nutritional status. Additional types of information that are relevant include level of nutrient in the background diet, methods used to estimate intake, analytical methods used to assess nutrient status, and whether a nutrient biomarker or other approach was used to validate the dietary data. Also, it is essential to document the food composition database used, and the algorithms used for estimation of content of nutrients in foods (Table 3).

**Table 3:** Diet assessment* (adapted from (3))

SI	Author Year Study Name	Exposure	Dietary assessment method	Food composition database definition of relevant nutrient	Internal calibration (or validity) of dietary assessment? (y/n). If yes, provide data	Biomarker Assay	Analytical validity of biomarker data reported? (y/n). If yes, provide data	Time between biomarker sampling and analysis	Season/Date when biomarker samples were drawn	Background exposure data
Clearly define foods, food groups, nutrients or other food components. When using dietary patterns or indices, describe the methods to obtain them. Specify if nutrient intakes are reported with or without inclusion of dietary supplement intake, if applicable			Refer to the name indicated in methods and specify the reference time (number of days recorded) and portion size estimation, if applicable. Report if and how supplement intake was assessed.	Indicate the full source of food composition database used to calculate dietary intakes (diet and supplements). Describe the use of conversion factors (weight yield and retention), if applicable.	Reference the validation study of the dietary or nutritional assessment method, including the reference method(s) used. The measures of validity should be reported (e.g. mean difference, correlation coefficient, classification agreement and limits of agreement).					Give the distribution of participant characteristics across the exposure variables. Provide an estimate for variation. Specify if food consumption of total population or consumers only were used to obtain results. Describe intake modeling (e.g. calculations of usual intake distributions) and use of weighting factors, if applicable.

* Write ‘nd’ if there was no data reported. Please do not leave blank.

## Step 6: Assessing risk of bias: critical appraisal of individual studies

The quality of the individual study will be appraised as internal validity, that is, how the study itself was performed and how this may have affected the results (Box 6). *Internal validity* refers to whether the study ‘correctly’ answers the research question (11, 20), that is, whether we can believe in the results. This is best done by assessing the RoB. The Cochrane collaboration defines *bias* as ‘systematic error, or deviations from the truth, in results or interpretation’, which leads to over- or underestimating the true intervention effect (20). As biased studies taken at face value will lead to misleading reviews, this is an integral aspect to consider. This should not be confused with lack of precision, which leads to random errors that can cancel out each other with sufficient replication.

Box 6 Key standards for assessing risk of bias

- Assess the risk of bias for each outcome to appraise the study’s internal validity, by at least two reviewers working independently.
- Use predefined risk of bias tools.

The assessment of RoB of individual studies is integrated with the later assessment of the total evidence (the strength of evidence).

### Sources of bias

The primary domains of bias are usually referred to as selection bias, performance bias, detection bias, attrition bias, reporting bias, and other biases (16, 21–23) (Table 4). It should be acknowledged that the same type of bias may underestimate the effect in one study and overestimate the effect in another study (20), and it may affect the estimate for one outcome but not another within the same study. Different types of biases may in theory even balance each other when it comes to the effect estimate if they work in opposite directions, but this is difficult to confirm (23).

**Table 4:** Types of bias

Main type of bias	Explanation	Issues
Selection bias – Bias arising from the randomization process	Assignment to intervention group is influenced by prognostic factors, leading to systematic differences in background characteristics between the groups being compared (i.e. confounding). Randomized, concealed allocation to groups uniquely limits selection bias.	Sequence generation (was the recruitment random?)* Concealed allocation (was the allocation of participants to groups concealed and unpredictable for participants and investigators?)* Control for confounding factors**
Performance bias – Bias due to deviations from intended interventions	Non-protocol interventions given, failure to implement the protocol, or non-adherence to the intervention by participants, due to awareness of intervention assignment.	Blinding of participants and investigators* Effect of assignment to intervention (‘intention to treat’) versus effect of adherence to intervention (‘per protocol’ effect).
Detection bias – Bias in measurement of the outcome	Measurement error or misclassification of outcomes. Causes bias if different between the groups.	Measuring methods appropriate? Blinding of outcome assessors Other potential threats to validity, for example, inadequate statistical analyses; exposure assessment method
Attrition bias – Bias due to missing outcome data	Systematic differences in attrition or length of follow-up of participants between groups, leading to incomplete outcome data.	Dropout or loss to follow-up Missingness is not by chance, but related to intervention group and the value of the outcome
Reporting bias – Bias in selection of the reported result	Systematic differences in what outcome measurement or analysis is reported and not.	Selective endpoint reporting (are any pre-specified or expected key outcomes not reported?)

* Not applicable to observational studies

** Applicable to observational studies

Although there is no general, empirically based standard for what degree of attrition/loss to follow-up is considered as low or high RoB, less than 20% dropouts from randomized controlled trials (RCTs) and above 50% participation rates in cohort studies may indicate low RoB in this domain. Significant differences (>20%) in dropout rates between intervention arms in RCTs conversely indicate high RoB. If one group has a higher dropout, and hence more missing outcome data, the estimated benefit of the intervention may be exaggerated if the attrition was due to the intervention.

The length of follow-up is more related to *indirectness* or *applicability*, which will be evaluated in the grading of the evidence, rather than a source of bias per se. Sample size is about power (e.g. if there is not enough power, the effects can be missed). Also, the sample size is not a source of bias but is related to *precision*.

Note that conflicts of interest or industry sponsoring of a study are not listed as criteria for bias as such, but the presence should be noted, and their possible influence on the RoB (especially selective endpoint reporting) should be considered (20, 23).

Bias assessments are often more complicated with non-randomized studies, such as prospective cohort studies. However, selection bias, performance bias, detection bias, attrition bias, and reporting bias should be assessed (13). Especially, the potential for selection bias is likely higher in most non-randomized studies such that the exposed and non-exposed groups are imbalanced when it comes to prognostic factors, leading to confounding (13, 24). Strictly speaking, confounding is not the same as bias, but it refers to internal validity, and it is important that these confounding factors (which should be listed in the protocol) are identified and to assess how they were managed. Note that controlling for variables that are intermediates in the causal pathway may bias the effect toward the null.

Thus, for non-randomized studies, confounding, selection bias, and misclassification bias due to methods used to ascertain exposure (i.e. dietary intake) and outcomes must also be assessed. In nutrition, the issue of exposure assessment is especially important to consider when deriving recommendations based on intake-response associations (25–27).

For randomized controlled trials, the RoB assessments are based on Cochrane’s ‘Risk of bias 2.0’ tool (20). The domains, and the criteria for judging low or high risk, are explained in the Appendix of the paper (20). Response options are Yes/Probably yes’, ‘No/Probably no’, or ‘No information’ (RoB 2.0 template: https://drive.google.com/file/d/18Zks7k4kxhbUUlbZ51Ya5xYa3p3ECQV0/view). A study may have low, some, or high risk for bias within each domain. The overall RoB per study is also judged as low, high, or unclear. Quotes from the paper or other sources of evidence for the judgment are put in tables. While NNR2012 used a three-category quality grading system (A, B, and C) similar to the Agency for Healthcare Research and Quality (AHRQ), SRs in NNR2022 classify the RoB in each outcome in each individual study into low, high, or ‘some concerns’ (21).

It should be acknowledged that a ‘low’ RoB judgment does not ensure that the study is totally free from bias, but that the risk is not so serious that it has any appreciable bearing on the results or conclusions. For instance, blinding is usually not possible in food-based dietary intervention, but this may not always imply a high RoB as long as it would not have influenced the outcome. Hence, the judgment of a study’s RoB as low, high, or unclear also depends on to what extent it is likely to affect the results.

For non-randomized trials, the assessment tool is based on the recent Risk of Bias in Non-randomized Studies of Interventions (ROBINS-I) instrument (21, 23). For observational studies (prospective cohort studies, case-cohort studies, or case-control studies), we will use the recently developed ‘Risk of Bias for Nutrition Observational Studies’ (RoB-NObS) tool developed by the US Department of Agricultur (USDA’s) Nutrition Evidence Systematic Review (NESR) team (28).

Although the interpretation and evaluation of RoB will involve some inevitable degree of personal judgment, a minimum of two independent reviewers will assess the RoB for at least one specific key endpoint within each study. If the two reviewers cannot resolve disagreements, a third reviewer will be involved. The assessment method should first be pilot tested with a handful of references to ensure mutual comprehension of the RoB criteria and questions.

**Fig. 2:**
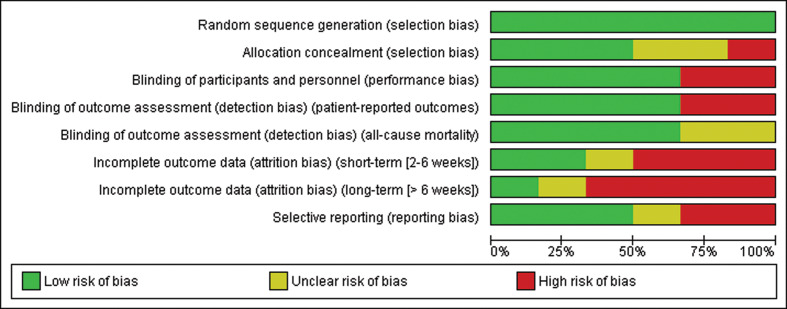
Example of a risk of bias graph figure, showing the proportion of studies with low, unclear, or high risk of bias. Reprinted from the Cochrane Handbook (20).

RoB tables and plots (see Fig. 2) must be presented in the final systematic review reports together with other study characteristics. Finally, an overall RoB for each main outcome across studies is reached to be included in the assessment of the strength of evidence. The RoB assessments will be used in the grading of the overall evidence. The study-level RoB will also be used to explore sources of between-study heterogeneity.

## Step 7: Synthesis and grading the strength of evidence

The evidence will always be synthesized qualitatively, in which the characteristics and context of the included studies, their strengths and limitations, heterogeneity (in study characteristics and results), and relevance, are reviewed and described (Box 7). The SR may also include a quantitative analysis, that is, a meta-analysis (see below), which should have been planned at the protocol stage. The meta-analysis results should be interpreted in light of the quality assessments.

Box 7 Key standards for synthesis and grading of evidence

- After the quality assessment of individual studies, the results of the quality assessment should be summarized, and the quality and strength of the evidence should be evaluated based on the summary of the results and quality assessment of all the individual studies.
- That is, how convincing is the evidence, taken the results from all the studies included in the review to support a judgment on a relationship.
- The expert groups grade the quality of a body of evidence separately for each outcome.

### Summary tables

The main results should be tabulated or listed in summary tables. A summary table is a distillation and synthesis of information from evidence tables. It is used to present study characteristics and results to support the interpretation of the evidence addressing a specific research question.

All the relevant outcome variables should be reported separately (e.g. morbidity, mortality, and biological effects). The outcome variables should include not only the positive effects but also possible negative effects (like elevated risk, side effects, and complications) and be presented separately in the summary table. The outcome variables should be tabulated hierarchically, with the more important variables presented before the less important ones. In some cases, also outcome variables with less relevance can be included but should be presented at the end of the summary table. Markers of study quality should be included.

Table 5 is an example of a summary table. There are a number of ways in which studies could be grouped for presentation. Grouping could be done according to the study design or other major factors that may influence the results. Forest plots of results can be used to illustrate the results of individual studies.

**Table 5:** Example on summary table headings

Exposure/intervention	No. of participants (No. of studies)	Outcome variable (primary or secondary)	RR (95% CI)	Effect	No. of studies rated as high/low/somewhat risk of bias	Strength of evidence (convincing, probable, limited –suggestive, limited – no conclusion)	Comments

In qualitative syntheses, it is not appropriate to simply tally or count the number of studies with positive and negative associations, or ‘significant’ results, as this does not account for, for example, lack of power or bias (29, 30). A ‘non-significant’ result in a study is also not evidence for no association/effect.

### Meta-analysis

Meta-analysis uses statistical methods to combine multiple studies addressing the same research question. It is often part of a systematic review and can identify significant results when individual studies are inadequately powered. Most meta-analyses combine weighted results across studies in order to arrive at an overall estimate. Effect estimates are typically expressed as absolute differences (risk difference), relative risk (risk ratio), or odds ratio for dichotomous outcomes (actually, the logarithmic RR and OR), or as weighted or standardized mean differences for continuous outcomes. Potential advantages of meta-analyses include an increase in power, an improvement in precision, the ability to answer questions not posed by individual studies, and the opportunity to settle controversies arising from conflicting claims. Still, the most important goal of a meta-analysis is actually to identify heterogeneity and patterns in the study results, rather than an average effect (29).

*Forests plots* can be used to illustrate estimates and confidence intervals for both individual studies and meta-analyses. Each study is represented by a block at the point estimate of intervention effect, with a horizontal line extending either side of the block. The area of the block indicates the weight assigned to that study in the meta-analysis, while the horizontal line depicts the confidence interval (usually with a 95% level of confidence). The area of the block and the confidence interval convey similar information, but both make different contributions to the graphic. The confidence interval depicts the range of intervention effects compatible with the study’s result and indicates whether each was individually statistically significant. The size of the block illustrates studies with larger weight (usually those with narrower confidence intervals), which dominate the calculation of the pooled result.

A technical statistical guidance for conducting meta-analyses is outside the scope of this handbook, and the review team (including statisticians) are referred to the most recent recommendations from AHRQ (31) and the Cochrane Handbook (30) in addition to textbooks.

Besides statistical measures, the diversity, sensitivity, and RoB in the evidence base must be considered before attempting to find a pooled effect (11). Pooling results from several studies with high RoB will give a correspondingly biased estimate even if the heterogeneity is low – ‘two wrongs do not make one right’.

Meta-analyses may therefore be conducted if it is considered appropriate to combine/pool the different studies. This cannot be objectively measured, but requires a judgment of clinical, methodological, and statistical heterogeneity (31). Sources of clinical heterogeneity include interventions/exposures, comparators, and participants, while methodological aspects include study design, type of outcome, and follow-up time. Both lead to statistical heterogeneity, that is, inconsistent directions or magnitudes of effects. If the included studies are so dissimilar in these aspects that an average effect will not be meaningful, then they should not be pooled and instead be reported with their individual results in tables and/or forest plots.

Other reasons not to pool study results include the existence of one or a few large studies that may be considered ‘best evidence’; wide-ranging effect estimates that indicate both beneficial and unwanted effects; suspicion of publication bias, and small-studies effects (see AHRQ’s guidance for further details (31)). Moreover, there should be caution about pooling only a small number of studies (e.g. less than 10 trials) and small sample sizes as the evidence may be underpowered, especially for rare outcomes such as total mortality in trials. In World Cancer Research Fund (WCRF), meta-analyses are only conducted when more than three independent RCTs of five cohort studies exist. The statistical heterogeneity may be underestimated when less than 5–7 studies are pooled (31).

Heterogeneity is especially important in syntheses and meta-analyses of non-RCTs because of many different methodologies. Fully adjusted effect estimates, not crude results, should be combined from non-RCTs, if otherwise is not justified. Different adjustment models can also be a source of potential heterogeneity. It is possible to do additional sensitivity analyses including minimally adjusted estimates, for example, to assess impact of confounding. PICO elements and the RoB domains should be assessed as potential sources of heterogeneity. It is critical that the possible impact of RoB is considered in the interpretation of the results, preferably formally by meta-regression analyses or subgroup analyses including only studies with low RoB.

When data are available, meta-regression should be performed to explain discrepancies across studies and to explore variations of effects, such as linear and non-linear dose–response relationships, although this must be interpreted in light of the ecological fallacy when pooling aggregated data (29). If subgroup analyses are undertaken, they should be predefined, and caution should be taken in the interpretation of subgroup differences. Interventional studies and observational studies should be meta-analyzed and reported separately.

### Strength of evidence

When the qualitative and/or quantitative syntheses have been performed, and before evidence-based recommendations can be developed, the overall strength of the evidence must be evaluated. The phrase ‘strength of evidence’ refers to the extent one can have confidence in that the effect estimate is correct. In general, this is affected by aspects of quality, quantity, and consistency in the body of evidence – specifically, the RoB, consistency/heterogeneity, and precision of the evidence are taken into consideration. Strength of associations, dose–response relationships, and supporting plausible evidence from experimental research are also important to assess when the evidence base includes observational research (32, 33). The grading is done per endpoint, but it is not necessary to grade the strength of evidence for every possible endpoint; only the most critical/relevant needs to be graded according to the purpose and the protocol.

#### Consistency

Consistencies in relative effect estimates, both effect size and its direction, should be assessed, and potential reasons for inconsistency should be examined, even when a quantitative synthesis is not performed. Understanding between-study variations may have important implications for giving recommendations.

#### Precision

Precision is concerned with the degree of certainty or uncertainty; it is largely a question of study size (number of subjects and events) and the confidence intervals around the effect estimate and is an indicator of random error. Less precise results will lead to a lower strength-of-evidence rating.

#### The World Cancer Research Fund criteria

The WCRF has developed a number of predefined criteria to grade the strength of evidence (Box 8) (33). These will be used in the *de novo* NNR2022 SRs.

Box 8 World Cancer Research Fund criteria

This box lists the criteria modified from World Cancer Research Fund (WCRF) that have been connected to the three-category quality grading system developed by the AHQR (34). The grades shown here are ‘convincing’, ‘probable’, ‘limited – suggestive’, and ‘limited – no conclusion’. It is important to note that the grades ‘convincing’ and ‘probable’ are both considered as strong evidence.

**Convincing (high)**

These criteria are for evidence strong enough to support a judgment that there is a convincing causal relationship or absence of relationship. A convincing relationship, or absence of relationship, should be robust enough to be highly unlikely to be modified in the foreseeable future as new evidence accumulates. All of the following criteria are generally required:

- Evidence from more than one study type (RCT, prospective/retrospective cohort, or case-control studies). For some outcomes (e.g. some risk factors), evidence from several RCT may be sufficient.
- Evidence from at least two independent cohort studies (cf. above).
- No substantial unexplained heterogeneity within- or between-study types or in different populations in relation to the presence or absence of an association or the direction of effect.
- Several good-quality studies with consistent findings to exclude with confidence the possibility that the observed association, or absence of association, results from random or systematic error, including confounding, measurement error, and selection bias.
- Presence of a plausible, biological gradient (‘dose response’) in the association. Such a gradient need not be linear or even in the same direction across the different levels of exposure, so long as this can be explained plausibly.
- Strong and plausible experimental evidence, either from human studies or relevant animal models, which typical exposures in humans can lead to relevant outcomes.

**Probable (moderate)**

These criteria are for evidence strong enough to support a judgment of a probable causal relationship. All the following criteria are generally required:

- Evidence from at least two independent cohort studies, or at least five case-control studies. For some outcomes (e.g. some risk factors), evidence from a few RCTs may be sufficient.
- No substantial unexplained heterogeneity between- or within-study types in the presence or absence of an association, or the direction of effect.
- Several good-quality studies (low risk of bias) with consistent findings to exclude with confidence the possibility that the observed association, or absence of association, results from random or systematic error, including confounding, measurement error, and selection bias.
- Evidence for biological plausibility, in case of an observed association.

**Limited – suggestive (low)**

These criteria are for evidence that is too limited to permit a probable or convincing causal, or absence of causal, relationship, but where there is evidence suggestive of a direction of effect. The evidence may have methodological flaws, or be limited in quantity, but shows a generally consistent direction of effect. All the following criteria are generally required:

- Evidence from at least two independent cohort studies or at least five case-control studies.
- The direction of effect is generally consistent though some unexplained heterogeneity may be present.
- Several studies of at least moderate quality.
- Evidence for biological plausibility.

**Limited – no conclusion (insufficient)**

Evidence is so limited that no firm conclusion can be made. A body of evidence for a particular exposure might be graded ‘limited – no conclusion’ for a number of reasons. The evidence might be limited by the amount of evidence in terms of the number of studies available, by inconsistency of direction of effect, by poor quality of studies (e.g. lack of adjustment for known confounders), or by any combination of these factors. Most of the studies have a high risk of bias or there are two or more low or moderate risk of bias (RoB) studies with contradicting or null results.

**Substantial effect on risk unlikely (strong)**

The evidence is strong enough to support a judgment that there is no substantial causal association between the exposure and outcome. This is unlikely to change in light of more studies in the near future.

All of the following are generally required:

- Evidence from more than one type of study design
- Evidence from at least two independent cohort studies
- A pooled effect estimate close to 1.0 in comparisons of high versus low exposure
- No substantial unexplained heterogeneity within or across study types or different populations
- Good-quality studies that exclude the possibility that the absence of association is because of random or systematic errors, including insufficient power, lack of precision or errors in exposure measures, too small range of exposures, confounding and selection bias
- Absence of a demonstrable biological gradient (dose-response)
- Absence of strong and plausible experimental evidence from human or animal studies

Some factors may warrant an upgrading of the strength of evidence:

- Presence of a plausible dose–response relationship between the exposure and outcome. This may not necessarily be linear (e.g. J- or U-shaped) or even in the same direction, as long as this can be explained plausibly. The absence of a dose–response relationship does not necessarily exclude any causal association.
- An especially large effect estimate (odds ration (OR) or relative risk (RR) ≥2, depending on the unit of exposure) after adjustment for confounding factors. This is less likely to be caused by bias due to confounding.
- Evidence from RCTs in humans.
- Evidence from well-controlled experiments demonstrating one or several plausible and specific mechanisms operating in humans.*
- Robust and reproducible evidence from experimental studies of appropriate animal models demonstrating that typical human exposures may cause the relevant outcome.

*This may also include Mendelian randomized studies when interpreting evidence from observational studies.

## Step 8: Reporting

The structure of the final report must adhere to the PRISMA reporting standards and as a minimum cover the following domains:

1. Title
2. Abstract
3. Introduction
  - Rationale
  - Objectives/research question (including PI/ECOTSS components)
4. Methods
  - Protocol and registration
  - Eligibility criteria (with explanations)
  - Information sources
  - Search
  - Study selection
  - Data collection process
  - Data items
  - Risk of bias in individual studies
  - Summary measures
  - Synthesis of results
  - Risk of bias across studies
  - Additional analyses
5. Results
  - Study selection (with flow diagram, and list of excluded studies assessed in full-text form)
  - Study characteristics (describe PI/ECOTSS components in detail)
  - Risk of bias within studies
  - Results of individual studies (presented both narratively and in summary tables)
  - Risk of bias across studies
  - Additional analysis
6. Discussion
  - Summary of evidence (including the strength of evidence)
  - Limitations
  - Conclusions
7. Funding and declaration of interest

See http://www.prisma-statement.org/ for further guidance.

The conclusions should summarize the evidence and should be clearly worded and based solely on the included evidence. They should also point out principal areas of uncertainty and areas where further research is required.
